# Effect of different pulse numbers of transcranial magnetic stimulation on motor cortex excitability: Single‐blind, randomized cross‐over design

**DOI:** 10.1111/cns.13248

**Published:** 2019-11-06

**Authors:** Zhi‐Ming Tang, Chun‐Yu Xuan, Xin Li, Zu‐Lin Dou, Yu‐Jie Lan, Hong‐Mei Wen

**Affiliations:** ^1^ Department of Rehabilitation Medicine The Third Affiliated Hospital of Sun Yat‐Sen University Guangzhou China

**Keywords:** excitability, motor cortex, pulse, repetitive transcranial magnetic stimulation

## Abstract

**Aims:**

We aimed to investigate the effect of different pulse numbers of high‐frequency repetitive transcranial magnetic stimulation (rTMS) over the motor cortex on cortical excitability in healthy participants.

**Methods:**

Fifteen healthy participants received 600 and 1200 pulses of 5‐Hz rTMS on separate days in a random order. Stimulation (duration, 2 seconds and interval, 1 seconds) was delivered over the left primary motor cortex for the hand, at 90% of resting motor threshold (rMT). The rMT and motor evoked potential (MEP) were measured before stimulation, and at 0 and 30 minutes after rTMS.

**Results:**

No significant differences were observed between the two conditions for MEP (*P* = .919) or rMT (*P* = .266). Compared with baseline, MEP was increased significantly at 0 (*P* < .001) and 30 minutes (*P* < .001) after stimulation. After stimulation, rMT was decreased at 0 minute for the 600 and 1200 pulse conditions (*P* < .001), but had recovered by 30 minutes (*P* = .073).

**Conclusion:**

Subthreshold 5‐Hz rTMS increased motor cortex excitability in healthy humans. However, the number of pulses may exhibit a ceiling effect in that beyond a certain point, that is, increasing the number of pulses may exhibit no further increase in cortical excitability.

## 1 INTRODUCTION

Transcranial magnetic stimulation (TMS) techniques can stimulate the cortical nerves noninvasively. The current generated by TMS can change the cell membrane potential of cortical nerves, causing a series of physiological and biochemical effects. Since it was first successfully applicated by Barker in 1985,^1^ TMS has been used successfully in the diagnosis and treatment of several neurological diseases.^2^, ^3^, ^4^

Transcranial magnetic stimulation could evaluate the excitability of corticospinal tract using the motor evoked potential (MEP) and resting motor threshold (rMT) parameters. Increase in MEP amplitude or decrease in rMT indicates improvement in excitability.^5^, ^6^

Repetitive TMS (rTMS) is the application of recurrent TMS pulses to continuously and repetitively stimulates the cortex. Certain parameters of rTMS modulate cortical excitability. rTMS frequency is the main factor that influences cortical activity. High‐frequency rTMS (≥5 Hz) increases cortical excitability, while low frequencies rTMS (≤1 Hz) decreases cortical excitability.^7^, ^8^, ^9^

Studies indicate that the number of pulses may also affect the regulation of brain excitability.^10^, ^11^, ^12^ Currently, however, there is no consensus on the optimal number of rTMS pulses required to achieve cortical excitability. Previous studies have used 150 to more than 2000 pulses for rTMS.^13^, ^14^, ^15^ Different rTMS pulse numbers might affect the changes in MEP amplitude and the effect duration. Increasing the pulse number within a limited pulse range, reportedly, has greater effects on excitability.^8^, ^10^, ^16^ However, it remains unknown whether a ceiling effect exists for increasing the numbers of rTMS pulses.

In this study, we investigated whether increasing the number of rTMS pulses could induce greater effects on cortical excitability. We compared the effect of 600 and 1200 pulses of 5‐Hz rTMS over the primary motor area responsible for hand control in healthy participants.

## 2 METHODS

### 2.1 Participants

Fifteen healthy college students were included in this study; seven were men and eight were women (Table 1), and their average age was 23.1 ± 1.2 years. All participants met the following criteria: (a) were right‐handed; (b) had no personal or family history of neurological or psychiatric disorders, including epilepsy; (c) had no pacemaker, electronic cochlea, or other metal implants or electronic devices; and (d) had no severe cervical disease including cervical instability. All participants who volunteered to participate in the experiment were informed of the experimental protocol and possible reactions and signed informed consent forms were obtained from them. This study was conducted in compliance with the Code of Ethics of the World Medical Association (Declaration of Helsinki) for experiments involving humans. Our study protocol was approved by Ethics Committee of the Third Affiliated Hospital of Sun Yat‐sen University.

**Table 1:** Subject's information and the order sequence

Subjects	Age	gender	Stimulate order	
			First pulses	Second pulses
1	23	W	600	1200
2	23	W	600	1200
3	25	W	600	1200
4	22	W	1200	600
5	22	M	600	1200
6	23	M	1200	600
7	22	M	1200	600
8	23	M	1200	600
9	24	M	600	1200
10	22	W	1200	600
11	25	W	1200	600
12	24	W	600	1200
13	21	M	600	1200
14	24	W	1200	600
15	24	M	600	1200

Abbreviations: M, Men; W, Women.

### 2.2 Study design

The study followed a single‐blind, randomized cross‐over design. All subjects received 600 and 1200 pulses of high‐frequency rTMS (CCY‐IA Wuhan Yiruide Co., Ltd.). The sequence of the 600 or 1200 pulses was randomly allocated according to computer‐generated numbers. The sequence number of each participant was enclosed in an envelope until the completion of rTMS.

### 2.3 Parameter settings for rTMS

Conditions for the application of 600 pulses were as follows: Frequency, 5 Hz; intensity, 90% rMT; pulse number, 600; pulse sequence, 60; sequence duration, 2 seconds; sequence interval, 1 seconds; and stimulation time, 3 minutes. The stimulation sites comprised specific areas of the left primary motor cortex.

Conditions for the application of 1200 pulses were as follows: Frequency, 5 Hz; intensity, 90% rMT; pulse number, 1200 pulses; pulse sequence, 120; sequence duration lasted 2 seconds; sequence interval, 1 seconds; and stimulation time, 6 minutes. The stimulation site comprised the left primary motor cortex area.

To avoid residual effects, the interval between the two stimuli conditions was longer than 24 hours. To avoid sequential effects, each participant received two stimuli in a randomized order (Table 1). The experimental duration was relatively fixed. The participants were asked to avoid consuming drugs or drinks that might affect their brain activity and to maintain a relatively regular schedule during the experiment period. The rMT was measured before stimulation, immediately after rTMS (0 minute), and 30 minutes after stimulation. To avoid tester bias, the rMT test before stimulation, and at 0 and 30 minutes after stimulation were performed by different testers. The second tester was blinded to the rTMS frequency.

The experiment was conducted in a quiet and comfortable environment. The participants were relaxed and sat on a chair with armrests on both sides. A pillow was placed over their thighs to relax their hands. The TMS coil's central point was fixed over the hotspot. The participants were instructed not to move their head to maintain a constant coil position during stimulation.

### 2.4 Evaluation parameters and assessment methods

Each participant's rMT was determined using a descending staircase method to find the lowest intensity at which five of the ten consecutive pulses applied induced the MEP amplitude greater than 50 µV.^17^, ^18^ The rMT was expressed as the percentage of TMS output intensity.

The procedure for measuring rMT was as follows: Participants’ bilateral upper limb muscles were kept relaxed, and the recording electrode was placed on the first dorsal interosseous muscle. The ground electrode was placed on the distal upper arm.

Determination of the stimulation hotspot: Hotspot was the position on the left primary motor cortex that could easily activate the first dorsal interosseous muscle. This point was the experimental location for measuring rMT and for delivering the stimulation. Using the 10‐20 electroencephalogram system, the C3 and C4 points were labeled on the scalp and 1‐cm points were marked around them. We stimulated the marked points initially with 70% of the TMS maximum output intensity. We determined the hotspot position by moving the coil to determine the location, which induced the biggest MEP amplitude on TMS with high repeatability. For measurement, the coil was placed in contact with the scalp, with the point aligned to the stimulus spot and the handle positioned at 45° to the middle line of the body.

Determination of rMT: In the relaxed state of the participants, we stimulated the hotspot with a high output intensity to induce MEP. The output intensity was reduced to identify the critical intensity, which on ten continuous stimulations could induce at least five MEP amplitudes greater than 50 µV. The output intensity was increased or decreased by 1% for fine‐tuning, and the above‐described process was repeated. The minimum stimulus intensity was considered as the rMT. The rMT was measured before rTMS, and at 0 and 30 minutes after rTMS.

MEP measurements were recorded from the first dorsal interosseous muscle by surface electromyography (Yiruide EE). We used TMS to stimulate the hotspot with 120% of the rMT intensity at the different time points.^16^ Five measurements were obtained at each time point.

### 2.5 Statistical analysis

We normalized the data of MEP and rMT at 0 and 30 minutes time points by the baseline data (value of time point‐value of baseline)/value of baseline. Data were represented as mean ± standard error (raw data and normalized data were shown on supplementary material). Repeated measures analysis of variance was used to analysis the difference of time points and conditions. We analyzed the interaction effects of condition factors (600 pulse vs 1200 pulse) and the time point factor (3 points) of MEP and rMT. Meanwhile, we analyzed the main effects of each factors. Then, we compared the mean differences between the three time points with the least significant difference method. Repeated measures analysis of covariance was used to analysis the orders effects. The data analysis was performed with SPSS 22 (IBM corporation.). The level of significance was designated as *α* = 0.05.

## 3 RESULTS

### 3.1 Changes in MEP

**Figure 1:**
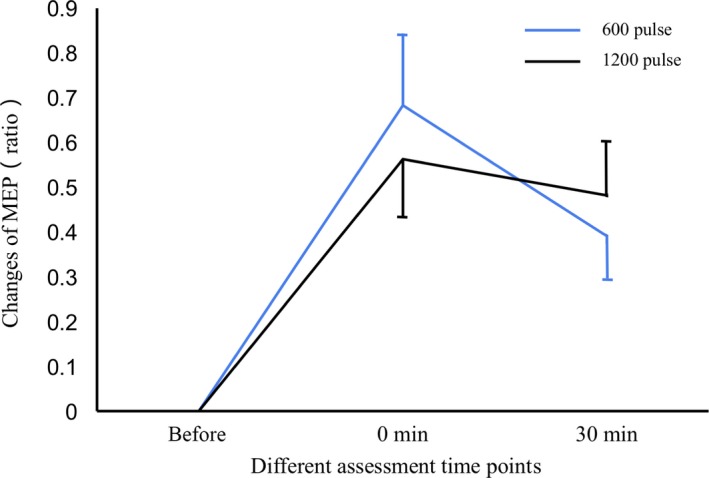
Changes in the motor evoked potential (MEP) before and after repetitive transcranial magnetic stimulation (rTMS). Changes in the MEP were similar in both conditions, rTMS with 600 and 1200 pulses (*P* = .919). MEP increased at 0 minute after stimulation (*P* < .001), and this increase persisted for 30 minutes (*P* < .001). Data were descripted as (Mean ± SE)

The results are shown in Figure 1. The different main time points had a significant effect on MEP (*F* = 24.097, *P* < .001). No significant main effect of condition was found (*F* = 0.010, *P* = .919). Two pulse conditions showed no interaction with the time points (*F* = 0.661, *P* = .501). Compared with baseline, MEP increased at 0 minute (*P* < .001) and decreased moderately at 30 minutes after stimulation. MEP after 30 minutes also differed statistically different from that at baseline (*P* < .001). No significant differences in MEP were found between the 0 and the 30 minutes time points (*P* = .059).

### 3.2 Changes in rMT

**Figure 2:**
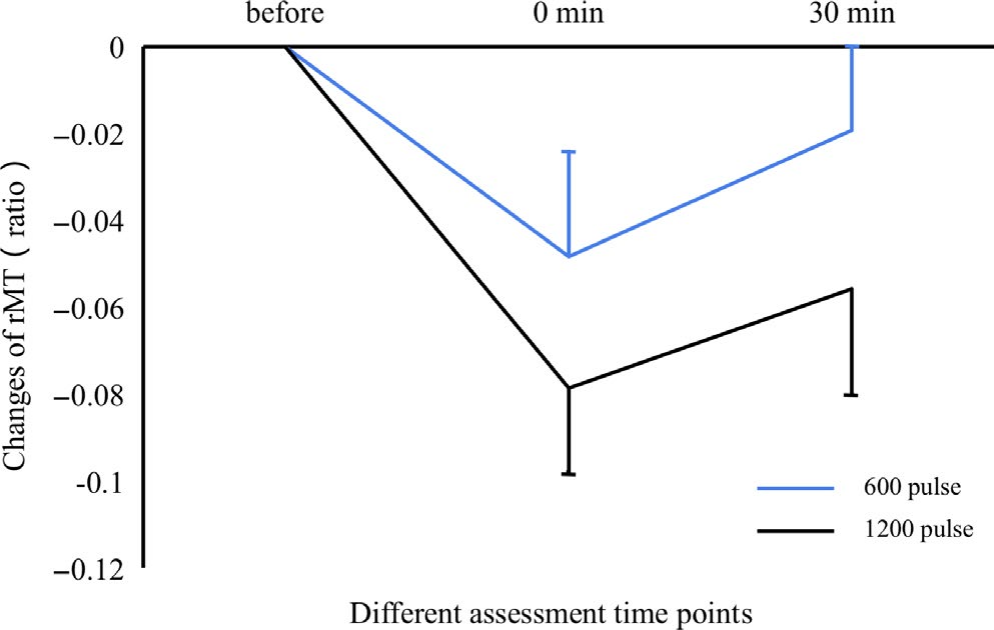
Changes in resting motor threshold (rMT) before and after repetitive transcranial magnetic stimulation (rTMS). Changes in rMT were similar in both conditions, rTMS with 600 and 1200 pulses (*P* = .266). rMT decreased at 0 minute after stimulation (*P* < .001) but recovered moderately by 30 minutes (*P* = .073). Data were descripted as (Mean ± SE)

The result was shown in Figure 2. rMT differed significantly between the three time points (*F* = 5.775 and *P* = .007). No significant main effect of condition was found (*F* = 1.286 and *P* = .266). rMT showed no significant interaction effect between the two pulse conditions and time points (*F* = 0.543 and *P* = .567). At 0 minute, rMT was decreased compared with that at the baseline (*P* < .001) and increased moderately 30 minutes after stimulation. However, the rMT at baseline and at 30 minutes after stimulation were not statistically different (*P* = .073).

### 3.3 Effect of orders

There was no significant main effect of orders found on MEP (*F* = 0.005 and *P* = .945) or rMT (*F* = 0.494 and *P* = .488). No significant interaction of orders and time points were found on MEP (*F* = 0.350 and *P* = .681) and rMT (*F* = 0.439 and *P* = .630).

### 3.4 Adverse reactions

Three participants experienced slight headache during rTMS. No other adverse reactions were reported.

## 4 DISCUSSION

This study investigated the effect of different pulse numbers of 5‐Hz rTMS on motor cortex excitability. Our results indicated that rTMS can improve motor cortex excitability; however, this excitability did not differ with 1200 pulses and 600 pulses of rTMS. This result indicated that a ceiling effect may be present for rTMS pulses numbers to induce cortical excitability.

Conventionally, a continuous pulse number is one of the most important parameters of rTMS. However, previous studies demonstrated no consensus on the optimal number of pulses to achieve cortical excitability. It remains unclear how the pulse number affects rTMS results. Nojima et al applied 1‐Hz rTMS with different pulse numbers (60, 120, and 240) over the right side motor cortex.^10^ Their results showed that MEP amplitude decreases with an increase in total pulse number, indicating that excitability, within a given range, increases with the number of stimulus pulses. Maeda et al applied 1‐ and 10‐Hz rTMS to the motor cortex with different pulses numbers (240 and 1600), at 90% rMT intensity.^12^ The MEPs reduced significantly after the 1‐Hz rTMS, while it increased significantly after the 10‐Hz rTMS. The magnitude of MEP decreased or increased significantly with pulse numbers, indicating that they affect the amplitude of cortical excitability. Peinemann's study found that 1800 pulses of subthreshold 5‐Hz rTMS induced a longer effect on corticospinal excitability than 150 pulses of the same stimulus.^11^ In our study, we compared the effect of 600 and 1200 pulses on corticospinal excitability. We found no effect on excitability with increase in pulse numbers. We predicted that the effect of rTMS would increase with higher pulse numbers in a certain range. However, a ceiling effect is observed after a certain threshold of pulse number has been attained.

In this study, 5‐Hz rTMS had a facilitatory effect on cortical excitability in healthy adults. Previous studies have also reported excitatory effects of rTMS, but the duration of excitation was unclear. For example, in Peinemann's study, the effect of 1800 pulses of rTMS lasted less than 40 minutes.^9^ Similarly, Muellbacher et al used 1‐Hz rTMS at 115% rMT intensity for 15 minutes and found significant reduction in motor cortex excitability for more than 30 minutes.^17^ In this study, the effect on MEP lasted at least 30 minutes. The effect on rMT did not last 30 minutes, although this may have been due to an insufficient sample size.

Presently, we used a cross‐over study design and randomized stimulus order on this study. The advantages of this design were that the same subject received two different types of stimuli, which can reduce the influence of different responses on different participants. We did the two stimulation conditions with an interval of more than 24 hours to avoid the possibility of sequential effects. Furthermore, we used a randomized order of stimulation to eliminate the interference of stimulation sequences. Finally, in this study no significant orders effect was found.

This study has several limitations. First, we only focused on the hand motor cortex area of the brain. It therefore remains unknown whether a ceiling effect exists for other brain areas. Second, the sample size was small, which might affect the efficiency of our testing. Third, the participants’ age was 20‐30 years, and the study included only healthy people. Fourth, we measured the rMT before measuring the MEP at each time points, which may affect the corticospinal excitability. Therefore, further experiments are needed with diagnosed patients in order to evaluate possible clinical applications.

In conclusion, 5‐HZ rTMS can significantly increase the corticospinal tract excitability, but the number of pulses may have a ceiling effect.

## CONFLICT OF INTEREST

The authors confirm that this article contains no conflict of interest.

## References

[1] Barker AT , Jalinous R , Freeston IL . Non‐invasive magnetic stimulation of human motor cortex. Lancet. 1985;1(8437):1106‐1107. 2860322 10.1016/s0140-6736(85)92413-4

[2] Chang WH , Kim YH , Yoo WK , et al. rTMS with motor training modulates cortico‐basal ganglia‐thalamocortical circuits in stroke patients. Restor Neurol Neurosci. 2012;30(3):179‐189. 22555430 10.3233/RNN-2012-110162 PMC3589123

[3] Zhang L , Xing G , Shuai S , et al. Low‐frequency repetitive transcranial magnetic stimulation for stroke‐induced upper limb motor deficit: a meta‐analysis. Neural Plast. 2017;2017:2758097. 29435371 10.1155/2017/2758097 PMC5756908

[4] Zhao N , Zhang J , Qiu M , et al. Scalp acupuncture plus low‐frequency rTMS promotes repair of brain white matter tracts in stroke patients: a DTI study. J Integr Neurosci. 2018;17(1):61‐69. 29376886 10.31083/JIN-170043

[5] Lanza G , Lanuzza B , Arico D , et al. Impaired short‐term plasticity in restless legs syndrome: a pilot rTMS study. Sleep Med. 2018;46:1‐4. 29773202 10.1016/j.sleep.2018.02.008

[6] Han T , Xu Z , Liu C , et al. Simultaneously applying cathodal tDCS with low frequency rTMS at the motor cortex boosts inhibitory aftereffects. J Neurosci Methods. 2019;324:108308. 31181244 10.1016/j.jneumeth.2019.05.017

[7] Gow D , Rothwell J , Hobson A , et al. Induction of long‐term plasticity in human swallowing motor cortex following repetitive cortical stimulation. Clin Neurophysiol. 2004;115(5):1044‐1051. 15066528 10.1016/j.clinph.2003.12.001

[8] Ling HM , Tao T , Xu J , Xu D . Effects of repetitive transcranial magnetic stimulation on upper limb motor function in patients with stroke: a meta analysis. Zhonghua Yi Xue Za Zhi. 2017;97(47):3739‐3745. 29325330 10.3760/cma.j.issn.0376-2491.2017.47.012

[9] Sasaki N , Abo M , Hara T , et al. High‐frequency rTMS on leg motor area in the early phase of stroke. Acta Neurol Belg. 2017;117(1):189‐194. 27502413 10.1007/s13760-016-0687-1

[10] Nojima K , Ge S , Katayama Y , Iramina K . Relationship between pulse number of rTMS and inter reversal time of perceptual reversal. Conf Proc IEEE Eng Med Biol Soc. 2011;2011:8106‐8109. 22256223 10.1109/IEMBS.2011.6091999

[11] Peinemann A , Reimer B , Loer C , et al. Long‐lasting increase in corticospinal excitability after 1800 pulses of subthreshold 5 Hz repetitive TMS to the primary motor cortex. Clin Neurophysiol. 2004;115(7):1519‐1526. 15203053 10.1016/j.clinph.2004.02.005

[12] Maeda F , Keenan JP , Tormos JM , et al. Interindividual variability of the modulatory effects of repetitive transcranial magnetic stimulation on cortical excitability. Exp Brain Res. 2000;133(4):425‐430. 10985677 10.1007/s002210000432

[13] Kondo T , Kakuda W , Yamada N , et al. Effects of repetitive transcranial magnetic stimulation and intensive occupational therapy on motor neuron excitability in poststroke hemiparetic patients: a neurophysiological investigation using F‐wave parameters. Int J Neurosci. 2015;125(1):25‐31. 24564818 10.3109/00207454.2014.897706

[14] Mally J , Dinya E . Recovery of motor disability and spasticity in post‐stroke after repetitive transcranial magnetic stimulation (rTMS). Brain Res Bull. 2008;76(4):388‐395. 18502315 10.1016/j.brainresbull.2007.11.019

[15] Gupta M , Rajak BL , Bhatia D , et al. Neuromodulatory effect of repetitive transcranial magnetic stimulation pulses on functional motor performances of spastic cerebral palsy children. J Med Eng Technol. 2018;42(5):352‐358. 30175934 10.1080/03091902.2018.1510555

[16] Yin Z , Shen Y , Reinhardt JD , et al. 5 Hz repetitive transcranial magnetic stimulation with maximum voluntary muscle contraction facilitates cerebral cortex excitability of normal subjects. CNS Neurol Disord Drug Targets. 2015;14(10):1298‐1303. 26556078 10.2174/1871527315666151111124216

[17] Ter Braack EM , de Goede AA , van Putten M . Resting motor threshold, MEP and TEP variability during daytime. Brain Topogr. 2019;32(1):17-27. 30019114 10.1007/s10548-018-0662-7 PMC6326963

[18] Zarkowski P , Navarro R , Pavlicova M , et al. The effect of daily prefrontal repetitive transcranial magnetic stimulation over several weeks on resting motor threshold. Brain Stimul. 2009;2(3):163‐167. 20161065 10.1016/j.brs.2009.02.001 PMC2747763

