# Supplementary material for: Effect of different pulse numbers of transcranial magnetic stimulation on motor cortex excitability: Single‐blind, randomized cross‐over design
Source: CNS Neurosci Ther. 2019 Nov 6;25(11):1277–81. doi: 10.1111/cns.13248 (PMC6834918; doi:10.1111/cns.13248)
Supplement: Supplementary file 1 [file CNS-25-1277-s001.docx]

Supplemental data

Table 1. Normalized MEP by baseline value of two conditions on different time points (mean ± SE)

| condition | baseline | 0 min | 30 min |
| --- | --- | --- | --- |
| 600 pulse | 0 | 0.683 ± 0.157 | 0.391 ± 0.093 |
| 1200 pulse | 0 | 0.563 ± 0.127 | 0.482 ± 0.117 |

Table 2. MEP amplitude of two conditions on different time points (mean ± SE, μV)

| condition | baseline | 0 min | 30 min |
| --- | --- | --- | --- |
| 600 pulse | 205.95 ± 16.99 | 282.69 ± 22.74 | 244.08 ± 17.66 |
| 1200 pulse | 220.72 ± 18.41 | 281.07 ± 22.73 | 268.8 ± 22.44 |

Table 3. Normalized rMT by baseline value of two conditions on different time point (mean ± SE, %)

| condition | baseline | 0 min | 30 min |
| --- | --- | --- | --- |
| 600 pulse | 0 | -0.048 ± 0.024 | -0.019 ± 0.032 |
| 1200 pulse | 0 | -0.079 ± 0.019 | -0.056 ± 0.024 |

Table 4. Raw rMT of two conditions on different time point (mean ± SE, %)

| condition | baseline | 0 min | 30 min |
| --- | --- | --- | --- |
| 600 pulse | 42.1 ± 3.46 | 39.6 ± 3 | 40.7± 3.06 |
| 1200 pulse | 43.4 ± 3.11 | 40.3 ± 3.28 | 41 ± 3.21 |
